# Grain Structure Engineering in Screen-Printed Silver Flake-Based Inks for High-Temperature Printed Electronics Applications

**DOI:** 10.3390/ma17204966

**Published:** 2024-10-11

**Authors:** Arjun Wadhwa, Mohammad Saadati, Jaime Benavides-Guerrero, Martin Bolduc, Sylvain G. Cloutier

**Affiliations:** 1 Department of Electrical Engineering, École de Technologie Supérieure, 1100 Notre Dame Street West, Montreal, QC H3C 1K3, Canada; arjun.wadhwa.1@ens.etsmtl.ca (A.W.); mohammad.saadati@etsmtl.ca (M.S.); jaime-alberto.benavides-guerrero.1@etsmtl.net (J.B.-G.); 2 Department of Mechanical Engineering, Université du Québec à Trois-Rivières, 555 Boulevard de l’Université, Drummondville, QC J2C 0R5, Canada; martin.bolduc2@uqtr.ca

**Keywords:** screen printing, high-temperature devices, silver flake ink, grain boundary pinning, printed electronics, silicon particles, electron backscattered diffraction (EBSD), X-ray photoelectron spectroscopy (XPS)

## Abstract

We extensively studied serigraphic screen-printed commercial silver flake inks loaded with silicon inclusions in order to achieve pinning at the grain boundaries. Based on grain size measurements using electron backscattered diffraction (EBSD), commercial silver ink with silicon microparticle content of 5 wt.% shows significant grain growth retardation compared to pristine silver ink, which stabilizes electrical conductivity up to 700 °C via a Zener pinning mechanism. The modified silicon-loaded silver ink experiences a two-times increase in grain size when heated up to 700 °C, compared to a seven-times increase for pristine silver ink. In turn, this enables operation temperatures significantly higher than the conventional operational window of microparticle-based silver inks, which are usually limited to 400 °C. Using isothermal exposures of 10 min up to 4 h, this phenomenon is observed at temperatures ranging from 250 °C to 900 °C. The electrical conductivity stability, grain size evolution and oxide contents were studied up to 4 h. The activation energy of silver ink with silicon inclusions is 54% lower than for pristine silver ink due to the pining effect, which retards grain growth via the Zener mechanism. Most importantly, the electrical resistivity remains stable up to 700 °C, which is more than twice the operation limit for off-the-shelf screen-printable silver flake inks. Hence, we demonstrate that adding controlled amounts of silicon particles to silver inks for grain structure engineering can open new vistas of possibilities for screen-printed metallic inks.

## 1. Introduction

In recent years, the field of all-printed flexible hybrid electronics (FHE) has rapidly advanced towards the creation of functional, flexible and low-cost devices for a variety of applications. Generally, FHE devices are limited in operational temperature by the thermal limits of the conductor (melting point) or polymeric substrate (glass transition temperature). In the context of high-temperature applications, FHE technologies have immense potential to deliver robust high-temperature sensors and power electronics [1]. Key industrial sectors such as aerospace, oil and gas and chemical processing have the opportunity to greatly benefit from the development of lost-cost strain, temperature and pressure sensors to monitor critical systems and processes [2]. Conventional conductive inks and substrates are generally unable to perform under conditions of elevated temperature exceeding 300 °C to 350 °C [3]. A robust and stable high-temperature FHE device can be achieved by optimizing its three main building blocks, namely its conductive ink, substrate and passivization overcoat material [4]. A successful high-temperature conductive ink should be able to maintain its high electrical conductivity, undergo minimal oxidation and remain stable over extended periods of time during its service life. Traditionally, high-cost and harder-to-process precious metal inks such as nickel, gold, platinum and palladium have been employed to make high-temperature sensors and devices [5,6,7,8,9].

Hence, there is an urgent need for low-cost and readily available inks that cater to high-temperature applications. To address this, significant effort has been invested into improving traditional conductive inks via two distinct strategies, namely the use of additives and alloying [1,4,10,11,12,13,14,15]. Passivating additives such as graphene have been used to minimize the oxidation of copper-based conductive inks [1]. Similarly, Ci–Ni, Ag–Cu, Cu–BN and Ag–Zn alloys have been successfully investigated for use in high-temperature printed device applications [4,10,11].

Silver inks remain the most widely used due to their high electrical conductivity, stability and low oxidation potential [16]. It is estimated that silver inks held a market share of over 21% in 2023 [17]. Relatively low costs and moderate sintering conditions make silver the material of choice for devices fabricated on low-temperature flexible polymeric substrates such as PET, PEN, polycarbonate and Kapton^®^ [18,19]. A large variety of silver inks is commercially available, depending on the intended printing method. Silver nanoparticle-based inks are used for direct-write printing such as inkjet, aerosol jet and micro-dispensing techniques [20], while silver microparticle inks are used for contact printing processes such as screen printing [21].

The sintering process and mechanism of silver particle-based inks have been detailed in the literature by several researchers [19,22,23,24,25,26,27]. The sintering process can be broken down into two stages, namely densification and grain growth. The optimal sintering temperature is reached when maximum densification is achieved while retaining the minimum grain size. This balance is challenging to achieve, since denisification leads to grain growth, as the two processes have the same driving force and mass transport mechanism. Additionally, grain growth is required to reduce interfacial energy, which eliminates pores and increases densification. Nano-sized particles tend to sinter at lower temperatures as compared to micron-sized particles [28]. Typically, silver nanoparticle inks achieve complete sintering at 300 °C (0.3 T_*m*_) [29]. Although microparticle screen-printable inks are designed for low-temperature substrates, they tend to achieve complete sintering at 400 °C (0.42 T_*m*_) [30]. Here, T_*m*_ is the is the melting temperature of bulk silver, which is 961 °C [31]. Beyond 0.42 T_*m*_, significant grain growth is expected, which in turn, alters the electrical properties and stability of devices [30]. Thus, the operational window generally acknowledged for screen-printed silver devices is up to 400 °C. Although this satisfies most applications, high-temperature FHE applications demand operation at temperatures exceeding 500 °C [2].

Grain growth is a well-known physical mechanism whereby the average grain size of a polycrystalline material increases when external energy is applied [32]. Grain boundaries are complex structures at crystalline domain interfaces typically 1–2 unit cells in width with higher Gibbs free energy than bulk crystalline material. When external energy is applied to polycrystalline material, grain boundaries move due to the diffusion of atoms from convex grains into concave grains, resulting in the grain boundary moving to the center of the concave grain. This is termed grain coarsening, leading to a reduction in grain boundaries and, hence, reducing the free energy of the polycrystalline material [33,34].

Electrical conductivity is largely dependent on grain size due to the presence of trapping sites at the grain boundaries, which influence electron mobility [35]. The electrical conductivity (σ) of nanoparticle-based inks is inversely proportional to the grain size [25,26], whereas the σ of microparticle-based silver inks is expected to increase with higher grain sizes up to 850 °C [28,30]. However, mechanical properties such as hardness and Young’s modulus tend to reduce with increases in grain size [27,32,33,36]. Here, it is imperative to find an optimal grain size to achieve desired mechanical and electrical properties of the printed film depending on the application at hand.

Building on the pioneering work initiated by Zener-Smith, it was shown that the inclusion of controlled amounts of secondary-phase particles help to achieve grain growth control [37]. This phenomenon is called grain boundary pinning or Zener pinning, whereby the dispersion of secondary-phase particles creates a pinning pressure that obstructs grain boundary movement to limit grain growth. Very limited work has been done to exploit such grain boundary control strategies using inclusions to engineer printable materials. Copper nanoparticles were previously alloyed with with gold [38], graphene [39] and silicon carbide [34] to achieve grain growth retardation, allowing for superior mechanical properties of the alloy for flexible printed device applications. Yttrium-stabilized zirconia was also added to silver ink, retarding grain growth up to 740 °C [40].

This study focuses on the process of grain growth and grain pinning of already sintered samples prepared as if they were to be used for ambient applications. Limited work is found in the literature on the electrical stability of printed silver inks at high temperatures. It has been reported that a significant increase in the grain size occurs for aerosol jet-printed silver ink at 500 °C, along with improved oxidation resistance and electrical stability at elevated temperatures [41]. Stability over long exposure durations is a critical parameter for the longevity and performance of high-temperature devices. This works seeks to provide a thorough understanding of the impact of refractory inclusions and how they can be used to control and improve the properties of commercial silver inks for operation at higher temperatures. Intrinsic silicon particles are used due to their low electronic band gap at room temperature, with 1.12 eV and a high electron mobility of 1350 cm^2^ (V s)^−1^ [42]. This allows for a smaller reduction in electrical conductivity of the modified ink as compared to more stable metal–oxide and carbide-based ceramics [42].

## 2. Materials and Methods

### 2.1. Materials

Widely used commercial screen-printable silver flake-based ink Metalon^®^ HPS-FG32 was purchased from Novacentrix Inc., Austin, TX, USA. The ink is 75 wt.% loaded with 1.5 μm silver flakes dispersed in a Butyl Carbitol-based solvent system. Silicon powder > 99% with particles 1–5 µm in diameter was purchased from US Research Nanomaterials Inc., Huston, TX, USA (product: US1127). In order to study the evolution of grain size at high temperatures, alumina substrates were employed due to their excellent high-temperature stability. Alumina sheets with a thickness of 0.2 inches were purchased from Mcmaster Carr, Elmhurst, IL, USA (product: 8462K26) and cut into 1 inch square coupons using a wet time cutting saw. No modifications were made to any of the materials upon receipt.

### 2.2. Ink Formulation

Modified (Ag–Si) ink was formulated by incorporating 3, 5, 7 and 10 wt.% loadings of Si microparticles into the pristine Ag ink. To achieve homogeneous dispersion, a two-step process was employed. The Si microparticles were weighed and added in approximately two portions. Each portion was added to the pristine Ag ink, followed by through mixing using a Thinky ARE-310 planetary mixer (Laguna Hills, CA, USA) for three cycles of one minute at 2000 rpm, followed by a one-minute rest interval to allow for ink degassing. This mixing procedure ensured a stable dispersion of the ink formulations, with a shelf life of over six months.

### 2.3. Sample Fabrication

**Figure 1:**
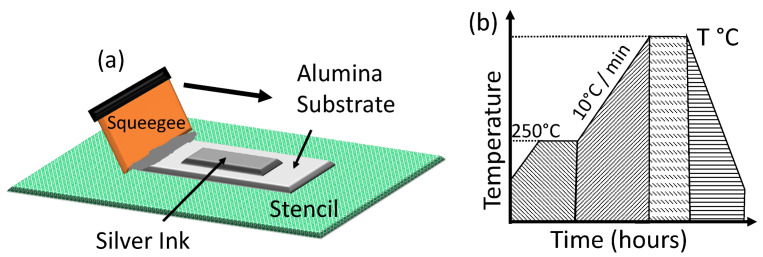
(**a**) Schematic of the screen printing process; (**b**) thermal cycling ramp profile.

Cut alumina substrates were prepared by cleaning with 9.99% pure acetone (Millipore Sigma, Burlington, MA, USA, product: 270725). A simple 17 mm × 12 mm rectangular feature was fabricated into a 325-mesh stainless steel mesh screen as depicted in Figure 1a, providing a 25.4 µm wet film thickness after manual screen printing (one pass), as seen in Figure 1a. Three samples per test temperature were fabricated for each of the modified (Ag–Si) formulations to ensure the repeatability and reliability of the collected data. As recommended by the silver ink manufacturer, the samples were sintered in a Mancorp MC301N reflow oven (Montgomery County, PA, USA) at 250 °C for 60 min in air to remove all organic components of the ink and initiate densification.

### 2.4. Testing and Characterization

We studied the evolution of the grain structure and electrical conductivity of the pristine Ag and (Ag–Si) inks with incremental temperatures and exposure times. The samples were exposed to temperatures in incremental steps of 100 °C with a ramp of 10 °C min^−1^ between 400 °C and 900 °C for 10 min and 1–4 h isothermal exposure times. The samples were then allowed to cool down naturally to room temperature. The thermal treatment was limited to 900 °C) to stay below the melting point of silver (961 °C) [43]. The entire thermal process is depicted in Figure 1b.

Raman spectra of the modified (Ag–Si) inks were obtained using a WITec alpha300A (Ulm, Germany) Raman microspectoscopy system with a 532 nm green laser. The particle size distribution of Si particles was determined using a Zetasizer Lab system (Malvern Panalytical Ltd., Malvern, UK). Micrographs of the printed films were obtained using a Hitachi SU8230 (Tokyo, Japan) scanning electron microscope (SEM) equipped with a Bruker QUANTAX FlatQUAD EDX detector (Billerica, MA, USA) for precise elemental mapping. The SEM was also equipped with a Bruker e-Flash HR+ detector to acquire electron back-scattering diffraction (EBSD) micrographs. An Ossila T2001A3 four-point probe (Sheffield, UK) was utilized to measure the electrical conductivity of the printed silver films. X-ray photoelectron spectroscopy (XPS) was performed using a Thermo Fisher (Waltham, MA, USA) VG ES-245 CALAB 250Xi equipped with a cobalt source. Thermal galvanometric analysis (TGA) was performed using a Perkin Elmer STA8000 system (Waltham, MA, USA).

## 3. Results and Discussion

### 3.1. Material Characterization

**Figure 2:**
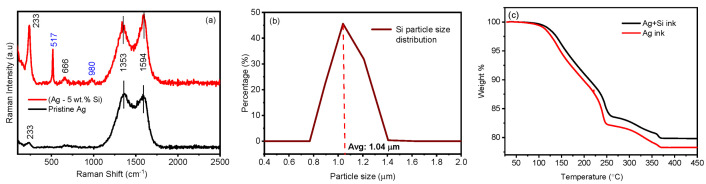
(**a**) Raman spectra of pristine and modified (Ag–Si) ink; (**b**) particle size distribution of Si nanoparticles as purchased; (**c**) thermogalvanometric analysis of pristine and modified (Ag–Si) ink.

The Raman spectra of the modified (Ag-5wt.% Si) is presented in Figure 2a). The band at 233 cm^−1^ is attributed to the Ag–O stretching mode [44]. The vibrational peak at 517 cm^−1^ is due to the stretching vibrations of C–N–C, and the peak at 666 cm^−1^ is due to the stretching vibrations of C–Si–C [45].

**Figure 3:**
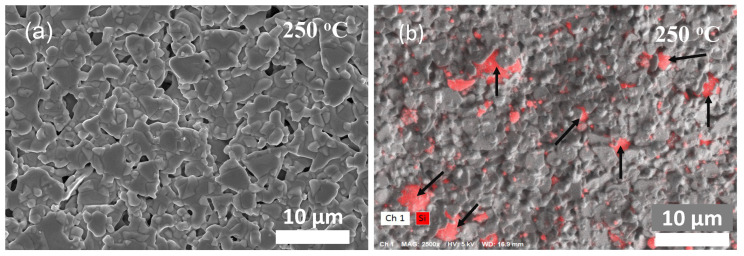
SEM micrographs of (**a**) pristine Ag ink and (**b**) EDX map of (Ag–Si) ink sintered at 250°C with Si particles (highlighted in red and black arrows).

The bands at 1353 cm^−1^, 1594 cm^−1^ are associated with the symmetric and anti-symmetric C=O stretching vibrations of the carboxylic group [46]. The peaks at 980 cm^−1^ and 517 cm^−1^ are from the silicon particles, which are slightly shifted as compared to crystalline silicon, with a peak at 520 cm^−1^ [47,48]. We further analyzed the particle size distribution of the as-received Si particles. Figure 2b suggests a size range between 800 nm and 1.4 μm, with an average particle size of 1.04 μm. Thermal galvanometric analysis (TGA) (Figure 2c) of both inks showed a significant drop in wt.% starting at around 120 °C until 250 °C, suggesting complete solvent burn-off, consistent with the boiling point of butyl carbitol at 231 °C. A further drop in wt.% between 250 °C and 366 °C can be speculated to originate from the removal of other organics in the ink matrix, such as proprietary surfactants and dispersants used by the ink manufacturer (Figure 2c). It is worth noting that the weight fraction of the modified (Ag–Si) ink is slightly higher than that of the silver ink, most likely due to the mass contribution of the silicon particles in the modified ink. The weight % of both inks remains stable beyond 400 °C, suggesting a complete sintering of the silver flakes beyond this point [49]. After initial sintering of the pristine Ag ink, at 250 °C (for 1 h), the silver flakes show signs of necking while maintaining their shape and structural integrity, indicating the onset of densification (Figure 3a). As seen in the EDX micrograph of the modified (Ag–Si) ink (Figure 3b), silicon particles (in red and highlighted with black arrows) are uniformly dispersed across the printed and sintered film.

### 3.2. Evolution of Electrical Conductivity

**Figure 4:**
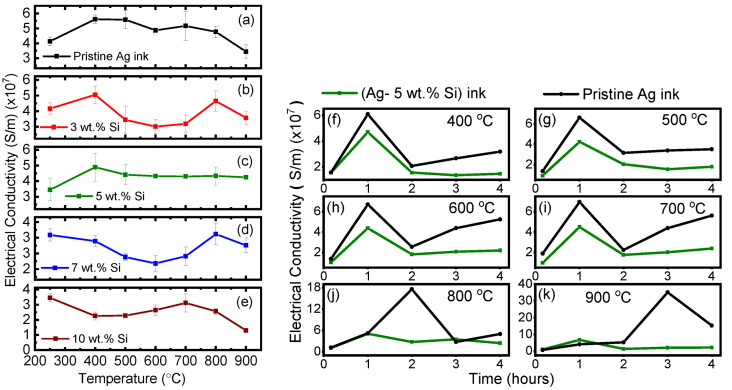
Electrical conductivity of (**a**) pristine Ag and modified (Ag–Si) inks with (**b**) 3 wt.%, (**c**) 5 wt.%, (**d**) 7 wt.% and (**e**) 10 wt.% silver inks treated at incremental temperatures for 1 h of isothermal exposure. Electrical conductivity of pristine Ag and modified (Ag-5 wt.% Si) inks thermally treated at (**f**) 500 °C, (**g**) 500 °C, (**h**) 600 °C, (**i**) 700 °C, (**j**) 800 °C and (**k**) 900 °C over 10 min and 1–4 h isothermal exposure.

Over the entire temperature range, we observed the impact of grain growth and grain pinning on the electrical conductivity of both inks that were studied. To study this effect, electrical conductivity was measured via a four-point probe for three samples processed at each incremental treatment temperature. Each sample was probed 3 times with a sample set of 100 data points per test. In total, 900 data points of electrical conductivity were collected per sample point. The evolution of electrical conductivity of the pristine Ag ink (1 h isothermal time per treatment temperature) is presented in Figure 4a. Conductivity data for all test cases are been summarized in Table S1 in the Supplementary Materials section. After initial sintering under the manufacturer’s recommended conditions, the pristine Ag ink achieved an electrical conductivity of 4.14 (±0.27) × $10^{7}$ s/m, which is approximately one-third that of bulk silver (6.2 × $10^{7}$ s/m) [50]. The conductivity further increased up to 500 °C, with a maximum value of 5.6 (±0.28) × $10^{7}$ s/m as grain growth continued, leading to a reduction in the mean free path and micropores, leading to densely packed film. Beyond 500 °C, we observed a fall in electrical conductivity owing to the formation of pores due to continued densification of the film’s microstructure.

Figure 4b–e show the evolution of electrical conductivity of the modified (Ag–Si) inks with 3, 5, 7 and 10 wt.% loadings of Si particles treated at incremental temperatures for 1 h of isothermal exposure. Here, we note that the conductivity of all the modified (Ag–Si) inks at 250 °C is lower than that of the pristine Ag ink. This reduction can be attributed to the presence of insulating Si particles in the inks. The 3 and 7 wt.% Si-loaded inks showed a initial increase in conductivity up to 400 °C, indicating complete sintering, followed by a reduction in conductivity due to increased grain growth. Beyond 700 °C, these inks behaved erratically.

The 10 wt.% Si loaded ink followed exhibited behavior; the conductivity reduced up to 500 °C, then tended to increase gradually until 700 °C, followed by a sudden drop in conductivity. The 5 wt.% Si-loaded ink was, by far, the most stable formulation. After complete densification was achieved at 400 °C, the electrical conductivity remained extremely stable up to 900 °C. This formulation has the potential to provide the optimal balance between densification and grain growth, allowing for stable electrical performance at ultra-high temperatures while maintaining a relatively low average grain size in the microstructure of the printed film, allowing for optimal mechanical properties according to the desired application. Next, we examine the properties of the modified (Ag-5 wt.% Si) ink as compared to the pristine Ag ink.

A robust and stable device needs to maintain its properties over a long duration of time, especially in extreme environments, where factors such as oxidation can play a detrimental role. To examine the electrical stability of the modified (Ag-5 wt.% Si) ink, we tested freshly prepared samples at each temperature for up to 4 h of isothermal exposure. Figure 4f–k compare the electrical conductivity of the pristine and 5 wt.% Si modified ink. Error bars are omitted for the simplicity of graphical representation; however, conductivity data with standard deviations are presented in Table S2 in the Supplementary Materials section.

For the modified (Ag-5 wt.% Si) ink, up to 400 °C, the electrical conductivity peaks at 1 h of isothermal exposure, which confirms the efficacy of the manufacturer’s recommended sintering conditions. Previous reports on microparticle-based silver inks have also described a rise in electrical resistivity between 350 °C and 450 °C after complete sintering has taken place [51]. Beyond 1 h, the conductivity of both inks remains stable and follows a similar trend up to 500 °C. Beyond 500 °C, the pristine Ag ink shows significant variability in its conductivity at longer exposure times, suggesting the transition to near-bulk-like silver due to rapid grain growth, followed by oxidation and melting of the silver ink as the temperature approaches 900 °C. These observations reinforce the practical notion that silver inks have an operational ceiling of approximately 400 °C for pristine silver ink. In contrast, the modified (Ag-5 wt.% Si) ink maintained stable electrical conductivity across all tested temperatures and prolonged exposure times. Although the conductivity of the modified ink is slightly lower, it has the potential to maintain a significantly stable grain size under the same test conditions, as explored in the next sections.

### 3.3. Evolution of Grain Size

**Figure 5:**
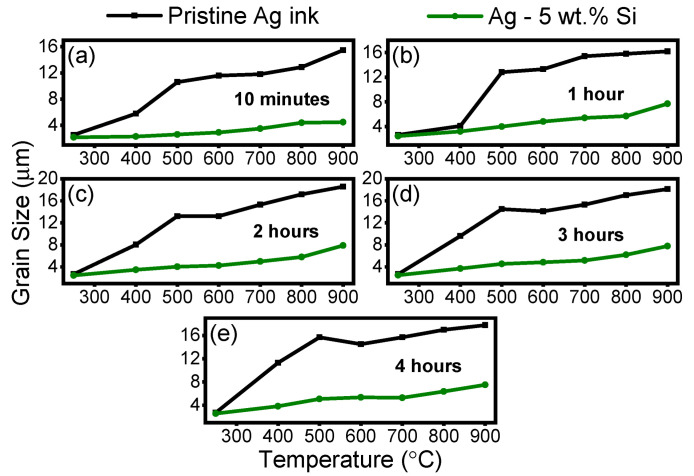
Grain size evolution of pristine Ag and modified (Ag-5wt.% Si) inks post thermal treatment between 250 °C and 900 °C for (**a**) 10 min, (**b**) 1 h, (**c**) 2 h, (**d**) 3 h, (**e**) 4 h of isothermal exposure.

After the thermal annealing tests, the morphology of the pristine Ag and modified Ag-5 wt.%Si inks were studied via scanning electron microscopy (SEM) and electron back-scatter diffraction (EBSD) analysis. Figure 5 and Table S3 (Supplementary Materials section) illustrate the evolution of the grain size for both the pristine and modified ink over the tested temperature range for each isothermal exposure time. It is to be noted that the grain size information for each sample was acquired via EBSD measurements. For nanoparticle-based inks, fast methods such as X-ray diffraction (XRD) analysis are simpler but are limited to measuring up to 200 nm grains [52]. In our case, the use of low-cost micron-sized flake-based ink required the use of EBSD analysis, which requires intense sample preparation via ion milling, leading to long and expensive experiments.

We observe a stark difference between the grain size achieved for the two inks for each isothermal exposure time over the entire thermal range. Interestingly, the pristine Ag ink sees a 7-times increase in grain size while the modified (Ag-5wt.% Si) ink sees a much lower 2.8-times increase in overall grain size between 250 °C and 900 °C for up to 4 h of isothermal treatment.

**Figure 6:**
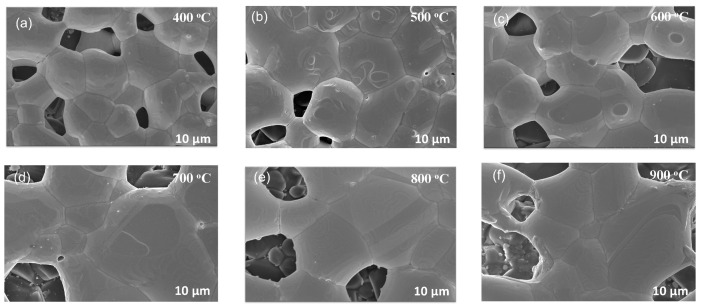
SEM micrographs of pristine Ag ink thermally treated at (**a**) 400 °C, (**b**) 500 °C, (**c**) 600 °C, (**d**) 700 °C, (**e**) 800 °C and (**f**) 900 °C for 1 h.

**Figure 7:**
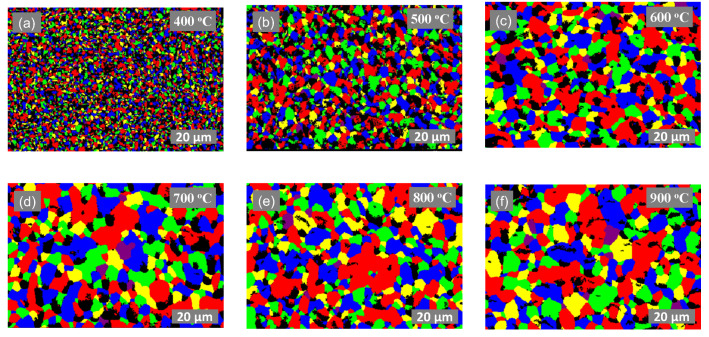
EBSD micrographs of pristine Ag ink thermally treated at (**a**) 400 °C, (**b**) 500 °C, (**c**) 600 °C, (**d**) 700 °C, (**e**) 800 °C and (**f**) 900 °C for 1 h.

The inhibition of grain growth is largely attributed to the presence of silicon particles in the ink, which facilitate Zener pinning. To further examine this phenomenon, we acquired SEM and EBSD micrographs of samples treated at each temperature interval. For the pristine Ag ink, Figure 6 illustrates microstructural changes with increasing temperatures, while Figure 7 illustrates the evolution of grain size for the same samples obtained via EBSD. The SEM micrographs indicate the formation of pores in the sample treated at 400 °C as densification accelerates, leading to increased grain size. At higher temperatures, smaller grains dissolve into larger grains, leading to a reduction in the mean free path, which, in turn, leads to an increase in electron mobility and electrical conductivity. However, pore size also increases at the same time, which is detrimental to the mechanical performance of the printed film [53]. EBSD micrographs confirm a significant increase in grain size, which can be attributed to aggravated growth and Ostwald ripening [51,54]. Grain growth by a factor of approximately seven is observed in the pristine silver ink at 900 °C. In Figure 6f, the pristine silver ink shows the transformation of micron-sized silver particles to a porous bulk-like solid film due to grain growth, reaching an approximate grain size of 18.2 μm after only 10 min of isothermal exposure. This is consistent with results seen in previous literature reports [55].

**Figure 8:**
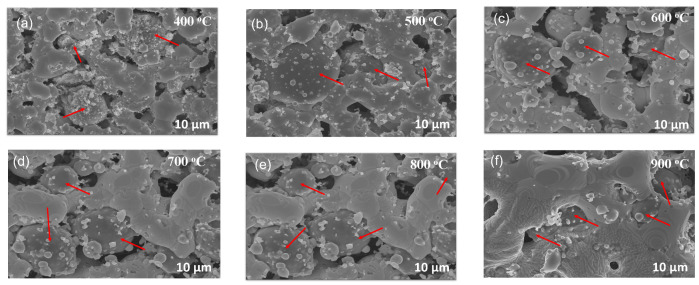
SEM micrographs of 5 wt.% modified (Ag-5 wt.% Si) ink thermally treated at (**a**) 400 °C, (**b**) 500 °C, (**c**) 600 °C, (**d**) 700 °C, (**e**) 800 °C and (**f**) 900 °C for 1 h.

**Figure 9:**
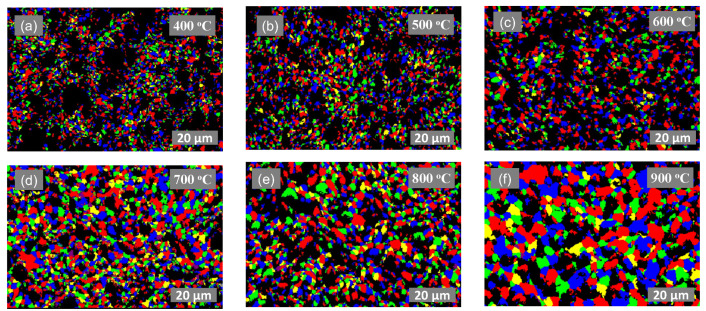
EBSD micrographs of 5 wt.% modified (Ag-5 wt.% Si) ink thermally treated at (**a**) 400 °C, (**b**) 500 °C, (**c**) 600 °C, (**d**) 700 °C, (**e**) 800 °C and (**f**) 900 °C for 1 h.

Figure 8 and Figure 9 exemplify the pinning nature of the silicon particles. Owing to the large particle size distribution in the purchased Si particles, we can segregate their impact into two main categories. First, we observe large Si particles (>1 μm) uniformly placed between segments of fused silver particles (highlighted with red arrows in Figure 8). These behave like pillars, physically preventing segments of silver particles from fusing together to form bulk-like material. Secondly, as the Ag grains grow, they encounter the smaller Si particles (400 nm–1 μm), which exert a drag force on the grain boundary, thereby impeding its movement. The drag force exerted by the Si particles on the grain boundaries is called pinning force, which stabilizes the grain boundaries and prevents their migration. In comparison to the pristine Ag ink, the modified (Ag-5 wt.%Si) ink exhibits an average grain size of only 4.5 μm when treated at 900 °C for only 10 min of isothermal exposure, which is less than one-quarter of the grain size observed under the same conditions for the pristine Ag ink.

**Figure 10:**
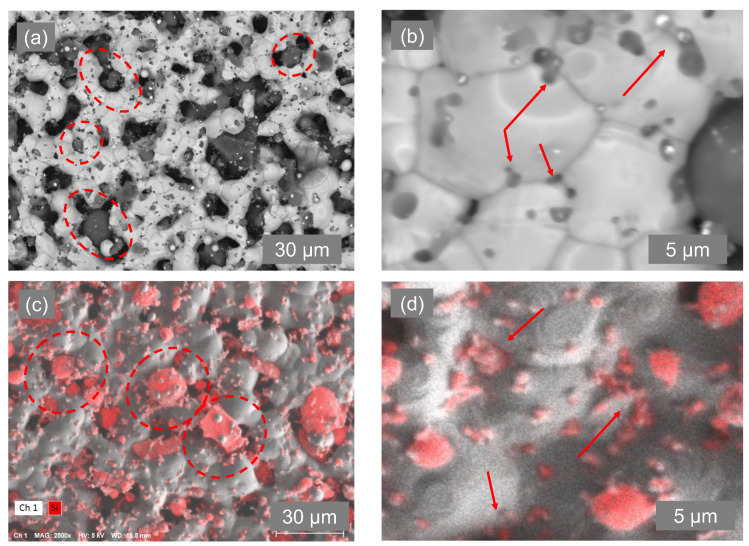
SEM and EDX micrographs exhibiting the effects of (**a**,**c**) large Si particles (highlighted with red circles) and (**b**,**d**) smaller Si particles (highlighted with red arrows) on the morphology of printed films.

We further confirm the effect of large and small Si particles in grain pinning in the modified (Ag-5 wt.% Si) ink, as seen in Figure 10. Figure 10a shows the presence of pillar-like Si particles evenly distributed within the silver matrix, retarding densification, whereas Figure 10b shows the presence of smaller Si particles that migrate to the grain-boundary interfaces, leading to Zener pinning. Figure 10c,d present the Energy-Dispersive X-ray (EDX) spectroscopy micrographs confirming the presence of Si particles as described (highlighted in red).

### 3.4. Oxidation States

**Figure 11:**
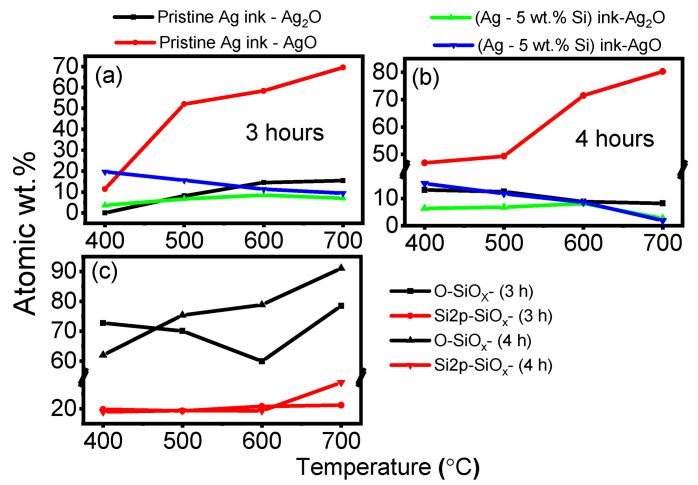
XPS analysis of the silver ink samples after annealing. (**a**) % Oxygen concentration in silver oxide species for the pristine silver ink samples after a 3 h of isothermal annealing; (**b**) % oxygen concentration in silver oxide species for the pristine silver ink samples after a 4 h of isothermal annealing. (**c**) % Oxygen in silicon oxide and total silicon oxide in the modified (Ag–Si) ink samples after 3 h and 4 h of isothermal annealing.

The ratios of the atomic wt.% of oxygen contributing to the silver and silicon oxide species were determined as shown in Figure 11 and summarized in Tables S4 and S5 in the Supplementary Materials section. For prolonged exposure times of 3 and 4 h, we observe an approximate 58% increase in the AgO (silver peroxide) species for the pristine Ag ink when heated from 400 °C to 700 °C due to the gradual oxidation of the silver particles (Figure 11a,b) [56]. This is consistent with reports of an operational ceiling of 400 °C for the pristine ink, beyond which the prints tend to oxidize [57]. On the other hand, the concentrations of both AgO and Ag_2_O species in the modified (Ag-5 wt.% Si) ink are significantly lower when treated at the same temperatures, and isothermal times and tend to decrease with increased temperatures. In addition, Figure 11c shows an increase in the amount of oxygen bonding to silicon, suggesting oxide formation becoming more pronounced beyond 400 °C [58,59]. X-ray photoelectron spectroscopy (XPS) spectra of the AgO and SiO_x_ species for temperatures ranging from 400 °C to 700 °C for 3 and 4 h of isothermal exposure are presented in Figure S1 in the Supplementary Materials section. Generally, silver oxide species begin to thermally decompose around 400 °C, at which point silver and oxygen are generated as per Equation (1) [60].

(1) $$2Ag_{2}O\left(s\right)\to 4Ag\left(s\right)+O_{2}\left(g\right)$$

This reaction involves the temporal formation of O before the formation of stable $O_{2}$, which results in increased oxygen presence. The Gibbs free energy change for oxide formation of Si is more negatively charged than Ag; hence, Si particles undergo preferential oxidation during this thermal cycling of the modified (Ag-5 wt.% Si) ink [61]. This reaction is depicted in Equation (2).

(2) $$2Ag_{2}O\left(s\right)+Si\to 4Ag\left(s\right)+SiO_{2}\left(s\right)$$

This process can be visualized as a scavenger effect, whereby the thermal energy from the furnace first decomposes the silver oxide species, creating free oxygen, which preferentially oxidizes silicon particles, thereby maintaining the integrity of the silver. We established that the improved stability of the modified ink is dominated by the following two mechanisms:

- The physical grain-pinning effect due to silicon inclusion ions present along silver grain boundaries, preventing silver domains from recombining (Zener pinning).
- The scavenger effect of the silicon particles to preferentially oxidize as opposed to silver particles, redirecting incoming thermal energy to the formation of silicon-oxygen bonds.

### 3.5. Grain Growth Kinetics

Grain growth kinetics is a well establish science pioneered in the 1950s [62]. Kinetics can be expressed as the n-power law shown in Equation (3) [63,64,65,66,67]. Here, D_*t*_ represents the average grain size at temperature T (in °C), and D_0_ represents the initial grain size. In our case, we consider D_0_ as the grain size at the manufacturer’s prescribed sintering temperature for the silver flake ink (250 °C). The term ‘t’ represents the exposure time in seconds, and ‘n’ is the kinetic growth-rate exponent. ‘k’ is the temperature- and activation energy-dependent proportionality constant, which can be calculated using Equation (4) [68,69,70], where k_0_ is the pre-activation constant, ‘Q’ is the activation energy in kJ mol^−1^ and ‘R’ is the universal gas constant.

(3) $$D_{t}^{n}-D_{0}^{n}=kt$$

(4) $$k=k_{0}exp^{\left(-Q/RT\right)}$$

Activation energy is the minimum energy required for electron flow within a material. A lower activation energy means that less energy is needed for these movements to occur. In larger grains, there are more interfaces for electrons to cross, leading to increased electron scattering and higher electrical resistance. As previously discussed (Figure 5), the pristine Ag ink experiences a significant increase in grain size over the entire exposure temperature and time range.

By adding a controlled amount of silicon particles to the silver ink (5 wt.%), a significant reduction in grain growth due to silver grain boundary pinning is observed. Thus, the grain growth equation can be modeled using Equation (5) [71,72] as follows:

(5) $$\frac{D_{0}-D_{t}}{D_{max}}+ln\left(\frac{D_{max}-D_{0}}{D_{max}-D_{t}}\right)=\frac{k}{D_{max}^{2}}$$

where $D_{max}$ represents the maximum grain size achieved at a given exposure temperature with an increase in exposure time. For the purpose of these calculations, we consider the grain size values obtained at 4 h of isothermal exposure as the $D_{max}$ values and the grain size at 250 °C as the $D_{0}$ value for each annealing cycle. The rate constant (‘k’) is calculated according to Equation (5) for 1 h of isothermal annealing, then fit using Equation (5). The kinetic growth-rate exponents are also calculated by fitting the experimental data, with values shown in Table 1.

**Figure 12:**
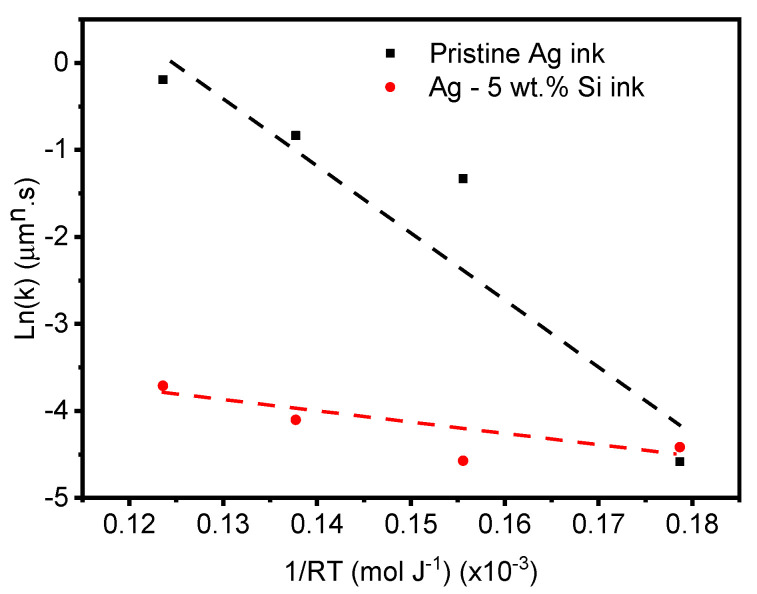
Arrhenius plot of the k parameter versus isothermal exposure temperature.

Generally, n = 2 is considered to correspond to normal grain growth controlled by curvature-driven grain boundary migration [73] and surface diffusion [55]. When n = 3 and higher, the grain growth is considered abnormal and can be attributed to a combined effect of solute precipitation, grain boundary grooving, impurity drag or volume diffusion along the grain boundaries [74]. By substituting the calculated values of ‘k’, we obtain the n values for both inks. Accordingly, the slope of the Ln(k) evolution as a function of the reciprocal of the isothermal temperatures ($\frac{1}{RT}$) directly provides the activation energies for each ink, as shown in Figure 12.

The pristine Ag ink experiences normal grain growth up to its optimal sintering temperature of 400 °C (n = 1.58), beyond which it follows an abnormal grain growth regime (n > 3) between 500 °C and 800 °C, which is in agreement with the results of other studies [75]. The activation energy is estimated to be 80.2 ± 19.15 kJ mol^−1^, which is in line with the reported activation energy of silver thin films of between 30 and 95 kJ mol^−1^ [55,63].

On the other hand, the modified (Ag-5 wt.% Si) ink exhibits normal grain growth (n < 3) over the entire temperature range. This is a strong indication of the pinning effect of the Si particles. The modified ink has a lower activation energy of 38 kJ mol^−1^, which is consistent with the impact of grain boundary pinning in microparticle systems [34,63,76].

When grain growth occurs at lower activation energies, it typically leads to a more controlled growth process, allowing for finer grains to form. This reduction in grain size means that charge carriers encounter fewer and less obstructive boundaries as they move through the material, thereby enhancing electrical conductivity. We observe that the electrical conductivity of the pristine Ag ink remains stable up to 400 °C, beyond which we see a steady decline in conductivity at higher temperatures as grain growth progresses. However, the modified (Ag-5wt.%Si) ink maintains a very stable conductivity at elevated temperatures and exposure times due to the Zener pinning effect.

The stabilization of the microstructure, electrical conductivity and oxidation of low-cost, screen printable silver inks opens up new possibilities in fabricating conductive electrodes and sensing devices such as silver RTDsfor high-temperature applications. Furthermore, the ease of the proposed modifications make this approach a viable option for large-scale production and deployment of sensors, which potentially alleviates the need for expensive and bulky sensors that are currently used for sensing applications in high-temperature environments.

## 4. Conclusions

The electrical stability of screen-printable silver ink for use at temperatures higher than 400 °C is poor. We have successfully demonstrated a silicon-based extrinsic grain boundary engineering process that offers benefits in minimizing grain growth while maintaining a high and stable electrical conductivity at significantly higher temperatures.

- Between 400 °C and 700 °C, the modified (Ag–Si) undergoes a 2× increase in overall grain size, exhibiting normal grain growth, whereas the pristine silver ink undergoes a 7× increase in grain size and exhibits abnormal grain growth.
- The electrical conductivity of both inks reaches a maximum at 1 h of isothermal exposure to each temperature point, indicating a transition point from sintering to grain growth. Between 1 and 2 h of exposure, the electrical conductivity reduces, indicating grain growth.
- Beyond 3 h, the pristine silver ink shows an erratic rise in electrical conductivity, indicating grain growth transitioning to the bulk material phase. This phenomenon is significant at higher exposure temperatures (800–900 °C).
- On the other hand, beyond 2 h, the electrical conductivity of the modified (Ag–Si) ink remains stable due to the Zener pinning effect.
- XPS data confirm a stark rise in silver oxide species in pristine silver ink with an increase in exposure temperatures, while the silicon particles in the modified (Ag–Si) ink preferentially bond with the oxygen, behaving like scavengers, thereby retarding the oxidation of the silver ink.
- The calculated activation energy for the modified (Ag–Si) inks is between 38 and 43 kJ mol^−1^, which is significantly lower than that for the pristine ink.

Using this strategy, the operational window of low-cost silver microparticle ink can be enhanced up to 700 °C, with stable electrical conductivity and minimal changes in its grain size. The authors intend to test the application of this modified ink as a functional material for high-temperature measurement in future work.

## Figures and Tables

**Table 1:** Calculations of n, k, activation energies (Q) and R^2^ values based on experimental results after 1 h of isothermal annealing at 400 °C, 500 °C, 600 °C and 700°C.

Temperature	Ag Only Ink		(Ag-5 wt.% Si) Ink	
	n	k	n	k
400 °C	1.58	0.010	2.41	0.012
500 °C	3.01	0.264	2.01	0.010
600 °C	3.57	0.433	2.20	0.016
700 °C	4.15	0.826	2.36	0.024
**Growth rate**	**Abnormal**		**Normal**	
Q (kJ mol^−1^)	80.2 ± 19.15		38.52 ± 7.345	
R^2^	0.9		0.93	

## Data Availability

The original contributions presented in the study are included in the article, further inquiries can be directed to the corresponding author.

## Supplementary Materials

The following supporting information can be downloaded at: https://www.mdpi.com/article/10.3390/ma17204966/s1, Figure S1: EBSD micrographs of 5 wt.% modified (Ag-5wt.%Si) ink thermally treated at (a) 400 °C, (b) 500 °C, (c) 600 °C, (d) 700 °C, (e) 800 °C and (f) 900 °C for 1 h; Table S1: Electrical conductivity data of pristine and modified (Ag–Si) inks with 3, 5, 7, 10 wt.% Si particles thermally treated for 1 h isothermal exposure at 250 °C, 400 °C, 500 °C, 600 °C, 700 °C, 800 °C, 900 °C; Table S2: Electrical conductivity data of pristine and modified (Ag-5wt.%Si) inks thermally treated between 250 °C and 900 °C for isothermal exposure times of 10 min, 1, 2, 3, 4 h; Table S3: Grain size evolution of pristine and modified (Ag-5wt.%Si) inks thermally treated between 250 °C and 900 °C for isothermal exposure times of 10 min, 1, 2, 3, 4 h; Table S4: Atomic wt.% of oxygen species observed via XPS analysis for samples treated for 3 h isothermal exposure; Table S5: Atomic wt.% of oxygen species observed via XPS analysis for samples treated for 4 h isothermal exposure.
