# Supplementary material for: Grain Structure Engineering in Screen-Printed Silver Flake-Based Inks for High-Temperature Printed Electronics Applications
Source: Materials (Basel). 2024 Oct 11;17(20):4966. doi: 10.3390/ma17204966 (PMC11509728; doi:10.3390/ma17204966)
Supplement: Supplementary file 1 [file materials-17-04966-s001.zip › materials-3209718-supplementary.pdf]

Supplemental Information

Table S1. Electrical conductivity data of pristine and modified $(Ag - Si)$ inks with 3, 5, 7, 10 wt.% Si particles thermally treated for 1 hour isothermal exposure at 250 °C, 400 °C, 500 °C, 600 °C, 700 °C, 800 °C, 900 °C.

**Temperature**	**Electrical Conductivity (S/m) ($x10^7$)**				
	**Pristine Ag ink**	**3 wt.% Si**	**5 wt.% Si**	**7 wt.% Si**	**10 wt.% Si**
250 °C	4.14 ± 0.27	3.83 ± 0.34	3.32 ± 0.22	3.46 ± 0.27	0.935 ± 0.34
400 °C	5.61 ± 0.27	4.65 ± 0.34	4.69 ± 0.685	3.46 ± 0.35	3.29 ± 0.34
500 °C	5.58 ± 0.28	3.17 ± 0.52	4.24 ± 0.866	1.96 ± 0.58	2.41 ± 0.31
600 °C	4.87 ± 0.61	2.76 ± 0.8	4.14 ± 0.63	2.79 ± 0.26	2.05 ± 0.18
700 °C	5.16 ± 0.41	2.93 ± 0.43	4.13 ± 0.22	3.12 ± 0.42	2.98 ± 0.43
800 °C	4.77 ± 0.96	4.28 ± 0.51	4.16 ± 0.93	2.41 ± 0.574	3.68 ± 0.51
900 °C	3.44 ± 0.4	3.28 ± 0.6	4.08 ± 0.56	1.29 ± 0.23	3.06 ± 0.6

Table S2. Electrical conductivity data of pristine and modified $(Ag - 5wt.\%Si)$ inks thermally treated between 250 °C and 900 °C for isothermal exposure times of 10 mins, 1, 2, 3, 4 hours.

**Temperature**	**Electrical Conductivity (S/m) ($x10^7$)**									
	**Pristine Ag Ink**					**Modified (Ag - 5 wt.% Si) ink**				
	**10 mins**	**1 hr**	**2 hr**	**3 hr**	**4 hr**	**10 mins**	**1 hr**	**2 hr**	**3 hr**	**4 hr**
250 °C	0.6 ± 0.085	2.9 ± 0.75	1.77 ± 0.14	2.08 ± 0.46	2.33 ± 0.76	0.825 ± 0.08	3.7 ± 0.4	1.12 ± 0.67	1.15 ± 0.12	1.12 ± 0.26
400 °C	1.58 ± 0.46	6.08 ± 0.88	2.08 ± 0.14	2.66 ± 0.46	3.18 ± 0.76	1.55 ± 0.54	4.69 ± 0.37	1.56 ± 0.67	1.36 ± 0.27	1.47 ± 0.26
500 °C	1.36 ± 0.25	6.62 ± 0.64	3.15 ± 0.85	3.38 ± 0.2	3.52 ± 3.60	0.925 ± 0.16	4.24 ± 0.88	2.04 ± 0.38	1.54 ± 0.27	1.79 ± 0.21
600 °C	1.37 ± 0.3	6.70 ± 0.82	2.55 ± 0.8	4.38 ± 0.41	5.24 ± 0.93	1.03 ± 0.34	4.39 ± 0.53	1.8 ± 0.13	2.06 ± 0.63	2.19 ± 0.6
700 °C	1.88 ± 0.62	6.96 ± 0.69	2.24 ± 0.465	4.38 ± 0.651	5.61 0.38	0.965 ± 0.3	4.50 ± 0.9	1.76 ± 0.23	2.01 ± 0.43	2.37 ± 0.44
800 °C	1.17 ± 0.153	5.21 ± 0.6	1.76 ± 0.389	2.77 ± 0.451	4.99 ± 0.67	1.07 ± 0.14	5.07 ± 0.556	2.77 ± 0.485	3.49 ± 0.41	2.41 ± 0.35
900 °C	6.92 ± 0.135	4.11 ± 0.44	5.26 ± 0.5	5.15 ± 0.41	5.26 ± 0.73	9.82 ± 0.18	6.76 ± 0.63	1.31 ± 0.293	2.04 ± 0.498	2.17 ± 0.146

Table S3. Grain size evolution of pristine and modified $(Ag - 5wt.\%Si)$ inks thermally treated between 250 °C and 900 °C for isothermal exposure times of 10 mins, 1, 2, 3, 4 hours.

**Temperature**	**Grain Size Evolution (μm)**									
	**Pristine Ag Ink**					**Modified (Ag - 5 wt.% Si) ink**				
	**10 mins**	**1 hr**	**2 hr**	**3 hr**	**4 hr**	**10 mins**	**1 hr**	**2 hr**	**3 hr**	**4 hr**
250 °C	2.7	2.64	2.67	2.7	2.72	2.16	2.44	2.48	2.51	2.56
400 °C	4.1	4.1	8.06	9.63	11.3	2.3	3.2	3.5	3.7	3.83
500 °C	12.8	12.8	13.2	14.5	15.7	2.6	4	4.03	4.55	5.08
600 °C	13.3	13.3	13.2	14.1	14.5	2.9	4.8	4.27	4.85	5.35
700 °C	15.4	15.4	15.3	15.3	15.7	3.5	5.4	5	5.18	5.28
800 °C	15.8	15.8	17.16	17.04	16.97	4.4	5.7	5.8	6.2	6.35
900 °C	16.2	16.2	18.56	18.135	17.768	4.5	7.7	7.9	7.8	7.5

Table S4. Atomic wt.% of oxygen species observed via XPS analysis for samples treated for 3 hours isothermal exposure

**Ag Ink**					**Modified (Ag+Si) Ink**				
**Species (%)**	400 °C	500 °C	600 °C	700 °C	**Species (%)**	400 °C	500 °C	600 °C	700 °C
O1s O in Ag2O	0	1.4	2.8	3.3	O1s O in Ag2O	1.5	2.9	3.9	3.3
O1s O in AgO	1.2	8.9	11.2	14.9	O1s O in AgO	8.1	6.8	5.2	4.4
O1s C-O-C, C=O	6	4.7	3.8	1.9	O1s O in SiOx, C=O, C-O-C	29.9	30.5	27.5	36.8
O1s O in C-O	3.3	2.1	1.4	1.3	O1s O in C-O	1.6	3.3	9.3	2.4
					Si2p in SiOx	19.8	19.3	20.8	21.2
**Total**	10.5	17.1	19.2	21.4	**Total**	41.1	43.5	45.9	46.9
% AgO	11.42	52.04	58.33	69.62	% AgO	19.70	15.63	11.32	9.38
% Ag_2O	0	8.18	14.58	15.42	% Ag_2O	3.64	6.66	8.49	7.03
					% SiO_x	72.74	70.11	59.91	78.46

Table S5. Atomic wt.% of oxygen species observed via XPS analysis for samples treated for 4 hours isothermal exposure

Ag Ink					Modified (Ag+Si) Ink				
**Species (%)**	400 °C	500 °C	600 °C	700 °C	**Species (%)**	400 °C	500 °C	600 °C	700 °C
O1s O in Ag2O	3.4	1.8	2	2.2	O1s O in Ag2O	2.7	3	3.7	1.6
O1s O in AgO	12.2	7.1	16.3	21.6	O1s O in AgO	6.6	5.2	3.9	1.1
O1s C-O-C, C=O	9	4	3.1	2.3	O1s O in SiOx, C=O, C-O-C	26.5	33.4	35.8	52.9
O1s O in C-O	1.4	1.5	1.4	0.8	O1s O in C-O	6.9	2.7	2	2.5
					Si2p in SiOx	19	19.4	19.3	28.8
**Total**	26	14.4	22.8	26.9	**Total**	42.7	44.3	45.4	58.1
% AgO	46.92	49.30	71.49	80.29	% AgO	15.45	11.73	8.59	1.89
% Ag_2O	13.07	12.5	8.77	8.17	% Ag_2O	6.32	6.77	8.14	2.75
					% SiO_x	62.06	75.39	78.85	91.04

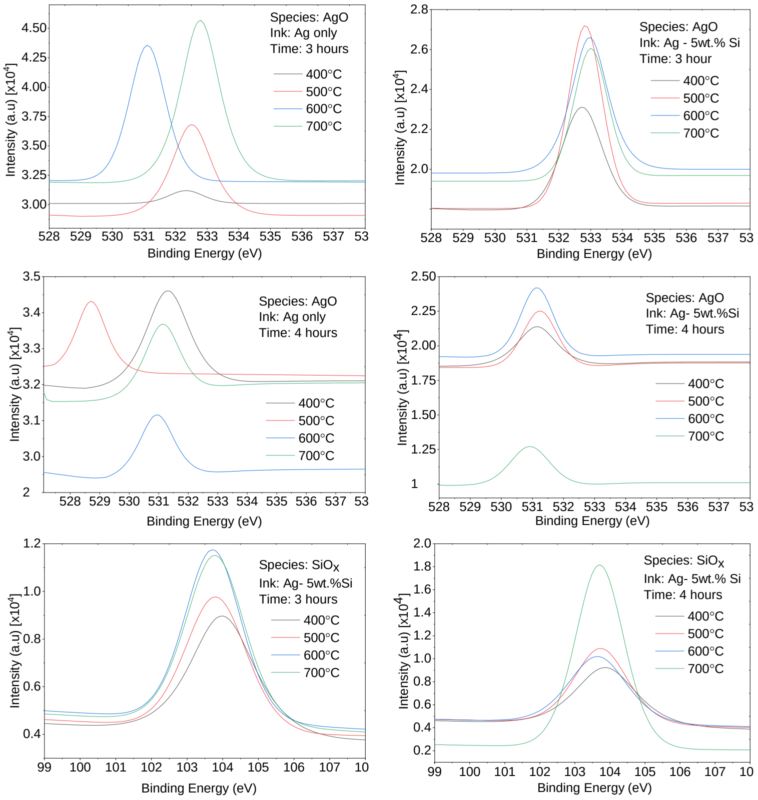

Figure S1. EBSD micrographs of 5 wt.% modified $(\mathrm{Ag} - 5\mathrm{wt.\%Si})$ ink thermally treated at a) 400 °C, b) 500 °C, c) 600 °C, d) 700 °C, e) 800 °C and f) 900 °C for 1 hour.
